# Supplementary material for: Temperature affects viral kinetics and vectorial capacity of Aedes aegypti mosquitoes co-infected with Mayaro and Dengue viruses
Source: Parasit Vectors. 2024 Feb 19;17:73. doi: 10.1186/s13071-023-06109-0 (PMC10877814; doi:10.1186/s13071-023-06109-0)
Supplement: Supplementary file 1 — Additional file 1: Figure S1. MAYV and DENV titers at initial infection (0 dpi) in adult and cell infection experiments. (a) Fully engorged female mosquitoes were collected whole at the sorting step as a proxy to determine the infectious viral particles ingested by each mosquito. (b) Viral inoculum delivered to the cells was tested after infection of each well. For both panels, each point represents a sample of an individual mosquito tissue or cell well. Purple shades depict MAYV titers and orange shades depict DENV. For co-infections, both viruses were tested on the same sample. [file 13071_2023_6109_MOESM1_ESM.docx]

# Supporting information


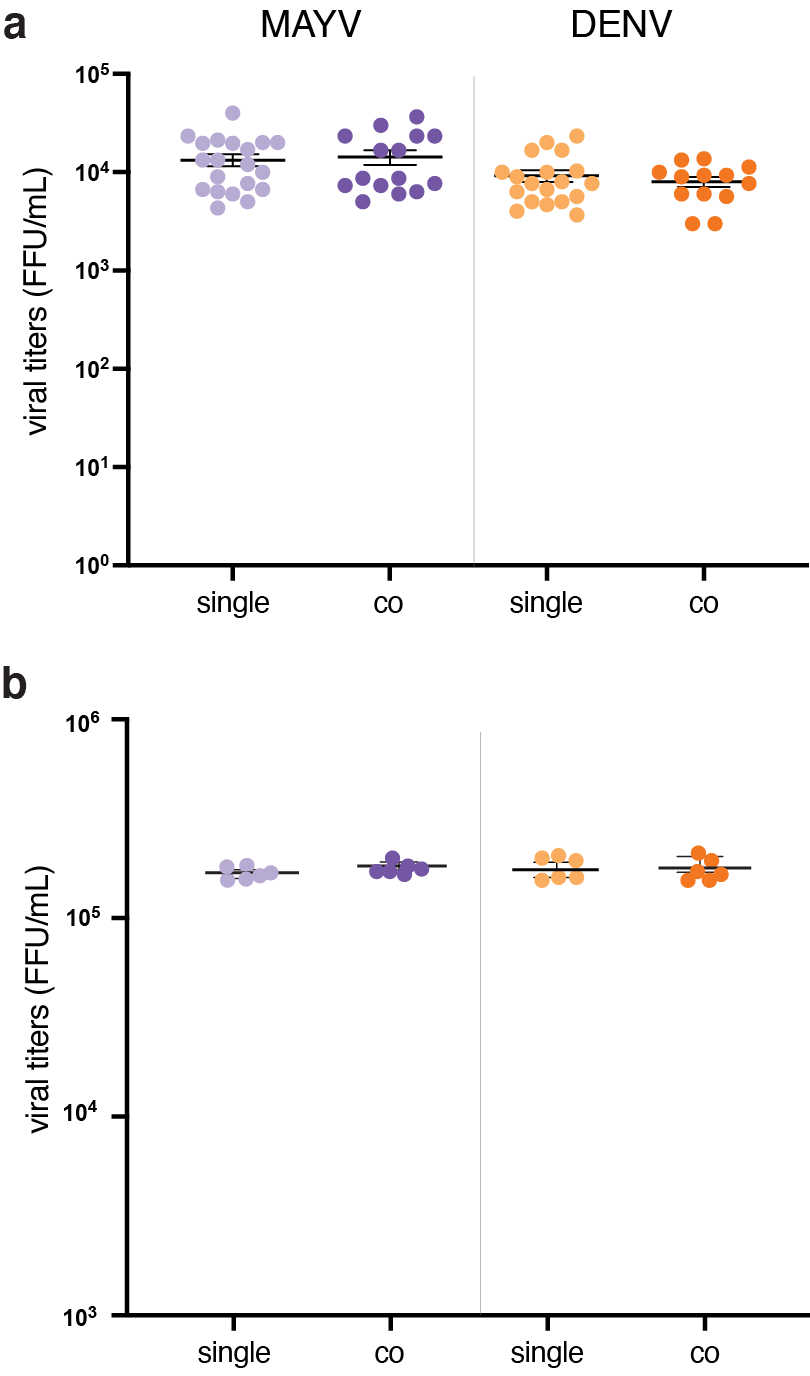


**S1 Fig. MAYV and DENV titers at initial infection (0dpi) in adult and cell infection experiments. a)** Fully-engorged female mosquitoes were collected whole at the sorting step as a proxy to determine the infectious viral particles ingested by each mosquito. **b)** Viral inoculum delivered to the cells was tested after infection of each well. For both panels, each point represents a sample of an individual mosquito tissue or cell well. Purple shades depict MAYV titers and orange shades depict DENV. For co-infections, both viruses were tested on the same sample.
